# A blistering new era for bullous pemphigoid: A scoping review of current therapies, ongoing clinical trials, and future directions

**DOI:** 10.1016/j.amsu.2021.102799

**Published:** 2021-09-04

**Authors:** Subaina Naeem Khalid, Zeest Ali Khan, Muhammad Hamza Ali, Talal Almas, Tarek Khedro, Vikneswaran Raj Nagarajan

**Affiliations:** a Shifa College of Medicine, Shifa Tameer-e-Millat University, Islamabad, Pakistan; b Royal College of Surgeons in Ireland, Dublin, Ireland

**Keywords:** Bullous pemphigoid, Primary blistering skin disorders, Treatment

## Abstract

Bullous pemphigoid (BP) is a severe autoimmune blistering skin disorder that primarily causes disease in the older population and is the most prevalent subepidermal variant of the pemphigoid diseases. It manifests as exquisitely pruritic vesiculobullous eruptions and is associated with significant morbidity and mortality. Studies are reporting an increase in prevalence, and, among the elderly, BP is no longer considered to be as rare as previously thought. The pathogenesis involves autoantibodies directed against proteinaceous components of hemidesmosomes, with consequent autoimmune destruction of the dermal-epidermal junction. In recent times, more complex elements of the underlying inflammatory orchestra have been elucidated and are being used to develop targeted immunotherapies. The primary treatment modalities of BP include the use of topical and systemic corticosteroids, certain non-immunosuppressive agents (tetracyclines, nicotinamide, and sulfone), and immunosuppressants (methotrexate, azathioprine, cyclophosphamide, and Mycophenolate). However, in the long term, most of these agents are associated with substantial toxicities while recurrence rates remain high. Such egregious prospects led to significant efforts being directed towards developing newer targeted therapies which work by attenuating specific newly discovered pillars of the inflammatory pathway, and these efforts have garnered hope in providing safer alternatives. Our review focuses on presenting the various therapeutic options that are currently in trial since December 2019, as well as on summarizing presently established treatment guidelines to provide readers with the latest exciting updates.

## Highlights

• Bullous pemphigoid (BP) is a severe autoimmune blistering skin disorder that primarily causes disease in the older population and is the most prevalent subepidermal variant of the pemphigoid diseases.
• In recent times, more complex elements of the underlying inflammatory orchestra have been elucidated and are being used to develop targeted immunotherapies.
• The present review focuses on presenting the various therapeutic options that are currently in trial since December 2019, as well as on summarizing presently established treatment guidelines to provide readers with the latest exciting updates.

## 1 Introduction

Bullous pemphigoid (BP) is one of the most prevalent subepidermal primary blistering disorders of the skin. It has a predilection for affecting elderly males, characteristically those over 70 years of age, and presents with blistering eruptions, tense pruritic bullae, and vesiculobullous dermatitis [1]. The global estimated disease incidence varies from 2.4 to 21.7 new cases/million inhabitants/year. In recent times, studies have reported worryingly increasing rates of prevalence [[2], [3], [4], [5]]. Additionally, the disease represents a significant burden on elderly individuals, with high healthcare costs and rates of mortality. Moreover, the prevalence increases substantially with age, and BP is no longer considered to be a rare dermatologic disease in elderly age groups [5]. (see Table 1)

**Table 1:** Clinical trials in bullous pemphigoid.

NCT Number	Intervention	Molecular Target	Masking	Phase	Status
NCT04612790	Benralizumab	IL-5R	Triple	3	Recruiting
NCT04563923	Avdoralimab	C5aR1	Open-Label	2	Recruiting
NCT04499235	AKST4290	CCR3	Double	2	Completed
NCT04465292	Tildrakizumab	IL23	Open-Label	1	Not yet recruiting
NCT04206553	Dupilumab	IL-4R/Il-13	Quadruple	2/3	Recruiting
NCT04128176	Rituximab combined with Omalizumab	CD20; IgE	Open-Label	3	Not yet recruiting
NCT04117932 v	Ustekinumab	IL-12/IL-23	Open-Label	2	Recruiting
NCT04035733	rVA576 (Coversin)	C5a-LTB4	Open-Label	2	Completed
NCT03295383	Rituximab	CD20	Triple	3	Recruiting
NCT03099538	Ixekizumab	IL-23/IL-17	Open-Label	2	Completed
NCT02226146	Bertilimumab	Eotaxin-1	Open-Label	2	Completed
NCT00525616	Rituximab	CD20	Open-Label	3	Completed
NCT01705795	Mepolizumab	IL-5	Double	2	Completed
NCT01688882	QGE031	IgE	Quadruple	2	Terminated
NCT03286582	AC-203	Inflammasome	Open-Label	2	Terminated

Some explanations for this increased disease burden among the elderly could include increasing life expectancy, increasing prevalence of disabling neurologic conditions, and increasing use of certain drugs (certain oral hypoglycemics, psychotropics, and monoclonal antibodies) [3]. Nevertheless, compounding evidence warrants scrupulous efforts be directed towards studying BP and raising awareness in order to curtail the growing burden of ailment. This review focuses on providing readers with a robust summary of the exciting new strides that have been made in the discovery of disease pathogenesis and how they have translated to the emergence of novel treatment strategies. While prior work summarizing potential agents is available [6], our review focuses on the therapeutic options that are currently in trial since December 2019, as well as on summarizing presently established treatment guidelines.

## 2 Pathogenesis

Before considering older and newer therapeutic agents, it is essential to review the relevant pathophysiology that these agents target, especially the latest strides that monastic study of the disease have yielded. Circulating autoantibodies that bind to proteinaceous components of hemidesmosomes cause BP. These components are vital to maintaining the functionality of the dermal-epidermal junction by attaching basal keratinocytes with the basement membrane.

Of particular importance are the two antigens BP230 and BP180, with the majority of patients having autoantibodies that adhere to the immunogenic extracellular domain (16A domain) of BP180. Self-reactive B and T cells against this 16A domain have also been found. Binding of autoantibodies (particularly IgG) to self-antigens ushers an inflammatory cascade which includes activation of the complement and coagulation cascades, recruitment of inflammatory cells, proteolytic destruction, endocytosis of antibody bound elements, and impaired adhesive function of hemidesmosomal proteins ending in tissue injury and creation of blisters [7,8].

The primary mechanisms that result in the generation of such autoimmunity are poorly understood and likely involve a mixture of hereditary predisposing factors and external influences, which result in the breakdown of immunologic tolerance to aforementioned self-antigens, eschewing the balance between autoreactive lymphocytes and regulatory lymphocytes. Some recognized risk factors include an HLA-DQβ1*0301 subtype and various factors that may play an inductive, facilitative, or contributive role. These include infections (e.g., CMV, Hep C, HHV-6, EBV, Pylori, and Toxoplasmosis), physical agents, and certain drugs (drugs that contain sulfhydryl groups e.g., penicillins and furosemide, drugs containing phenol rings e.g., aspirin and cephalosporins, as well as TNF-alpha blockers). While it is important to take note of these various factors, a vast majority of disease remains idiopathic [9]. Associations with Alzheimer's disease, multiple sclerosis, vitamin D deficiency, and thrombosis have also been suggested [10]. For the longest time, this baseline simplified understanding persevered, while the more complex components pillaring this inflammatory orchestra remained elusive.

Recently our understanding of these components has evolved, unearthing previously obscure mechanisms. One such significant revelation was involvement of the IgE, eosinophil, and mast cells axis. Prior work had already established eosinophilic presence within lesions and peripheral eosinophilia as a characteristic feature of BP. However, circulating anti-BP180 IgE immunoglobulins, coupled with eosinophils, are now thought to play a vital part in autoimmune damage as eosinophils are abundantly found within lesional fluid along with major basic protein [[11], [12], [13], [14], [15], [16], [17]]. Lin et al. recently demonstrated in an animal model that the attachment of anti-BP180 IgE to basal keratinocytes recruited eosinophils via interaction with the FcεRI receptor. Eosinophil degranulation, tissue damage, and blister formation followed shortly thereafter [12,18]. It has also been shown that amounts of anti-BP180 IgE in the serum are associated with the severity of disease and may be used to guide therapeutic decisions [[19], [20], [21]].

Closely knit to the pathomechanisms of humoral immunity described above are cell-mediated responses and the cytokine profiles they elaborate. A recently published meta-analysis by Kowalski et al. showed that BP patients had severely deranged levels of several cytokines including CCL-2, CCL-17, IL-5, IL-6, IL-8 and IL-17. It also described increased blister fluid levels of CCL11, eotaxin, and TNF-α [22,23]. Reports also indicate elevated expression of IL-18 and NLRP3 inflammasome components within mononuclear cells of individuals suffering from BP [24]. Jan et al. also delineated interleukins 17 and 23 as essential factors that favored the expression of IL-1β in macrophages collected from BP patients, with IL-1β driving inflammasome activation [25]. Finally, studies suggest the involvement of enhanced Th17 cell-related inflammatory processes and reduced Treg-related regulatory functions [26]. Each of these elements may represent targets for future therapeutic interventions.

## 3 Current treatments

### 3.1 Systemic corticosteroids

Historically, systemic corticosteroids served as the mainstay of bullous pemphigus treatment for more than 40 years despite their adverse effects and higher chances of relapses [27,28]. Oral prednisolone (PSL) at concentrations of 0.5–1 mg/kg/day represents the most widely accepted regime for effective disease control [[29], [30], [31]]. However, daily systemic corticosteroid quantities that were more than 0.75 mg/kg showed association with significant mortality [32].

In a recent clinical trial, the records of 78 BP patients were reviewed [33]. Of these individuals, 49 (62.8%) were administered oral prednisolone (PSL) while 29 (37.2%) were treated without PSL. The older patients with a lower Bullous Pemphigoid Disease Area Index (BPDAI) and/or lower anti-BP180NC16a antibody titer at the start of the disease were preferably treated without oral PSL. BP patients with PSL were given 0.1 mg/kg and 36.7% of them experienced a relapse. Amongst those who were not given oral PSL, only 9.1% of the patients faced relapse; thus, milder forms of BP are more effectively treated without doses of oral PSL.

On the other hand, fairly aggressive management with intravenous methylprednisolone 750–1800 mg/day in eight patients of the older age group exhibited decreased blistering in 24 h. However, this was at the cost of subsequently increasing morbidity risk [32,34].

The most frequently encountered side effects of systemic steroids include elevations in blood pressure levels and weight gain. Long-term drug usage is also affiliated with the development of diabetes mellitus, infections, Cushing syndrome, adrenal suppression, peptic ulcer, osteoporosis and proximal myopathy [35,36].

The relative contraindications to CS therapy include severe osteoporosis, diabetes mellitus, pseudotumor cerebri, aplasia, psychosis, steroid induced myopathy, and increased intraocular pressure. Among the absolute contraindications, herpes simplex ocular infection and active tuberculosis are notable [36].

### 3.2 Topical corticosteroids

Topical steroids have been recommended as a first-line agent according to data gathered by the Cochrane review and European guidelines [29,37]. Based on trials and retrospective studies on a total of approximately 800 participants, it was concluded that potent topical CS treatment was the most effective during an acute stage of BP [29,[38], [39], [40]]. It was also determined that, in comparison with oral prednisolone, doses of topical CS improved survival time and provided more efficacious results for BP treatment [39,41].

According to the Clinical Practice and Guidelines of Bullous Pemphigoid, topical steroids are recommended at a dosage of 0.05% Clobetasol propionate cream 10–40 g/day [36]. Topical corticosteroids as compared to oral corticosteroids proved to be more beneficial for treating patients with extensive BP in comparison to the patients who had moderate bullous pemphigoid [42].

In a comparative study between the standard topical corticosteroid dose (40 g/day) and the mild dosage (10–30 g/day), results suggested that individuals who suffered from moderate BP benefited from a mild regimen [35]. There was also a prominent decrease in the probability of serious adverse events with the mild dosage treatment. There were a few contraindications for the usage of topical corticosteroids for BP which included; active skin infections, increased sensitivity to the active component, rosacea, perioral dermatitis and acne vulgaris [36].

Adverse effects associated with topical use of steroids include telangiectasia formation, skin atrophy, striae, hypertrichosis, local super-infection. A rare side effect of topical therapy is hypersensitivity due to systemic absorption of the drug, resulting in side effects compatible with those due to systemic drug therapy [36,43].

### 3.3 Non-immunosuppressive agents

In case of partial or no response to steroid therapy, non-immunosuppressive agents are recommended as an adjuvant treatment. Tetracycline and nicotinamide possess anti-inflammatory functions, which are useful in the treatment of mild-moderate BP [36,44]. These treatments have relatively fewer side effects and hence are excellent treatment options for children and older age groups. Tetracyclines attenuate chemotaxis of neutrophils and eosinophils along with reduction in enzymatic destruction caused by matrix metalloproteases. A combination of tetracycline with nicotinamide is often used, which itself affects various aspects of the inflammatory response. It has been proposed that nicotinamide behaves as an electron scavenger, phosphodiesterase inhibitor, and it stimulates the conversion of tryptophan to serotonin [45,46]. However, when the effectiveness of prednisolone versus tetracycline and nicotinamide therapy was compared, there was no significant difference between the two regimens [36,47,48].

The dose recommendations for BP treatment include: tetracycline (0.5–2 g/day), doxycycline (200–300 mg/day) and nicotinamide (500 mg-2.5 g/day). The adverse effects of tetracyclines include renal insufficiency and potential liver failure in children of ages less than 12 years.

Another notable drug in the treatment of BP is sulfone. It has been shown to inhibit neutrophilic binding to the vascular endothelium thereby reducing chemotaxis, and the activity of myeloperoxidase (neutrophil/eosinophil derivative). Its application in treating BP has been demonstrated by numerous retrospective studies, reporting response rates ranging from 15% to 45%. A key obstacle associated with sulfone therapy is the development of anemia, which can develop even at therapeutic doses. It can also cause dose-dependent methemoglobinemia. The recommended starting dose of Sulfone is 50 mg/day and the maintenance dose is 100 mg/day. For children the dosage recommendation is 0.5–2 mg/kg/day [36,49,50].

### 3.4 Immunosuppressive agents

Among the immunosuppressive agents, methotrexate is considered the first-line drug and is used as monotherapy or in adjunct to the conventional topical corticosteroid treatment for BP [51]. This treatment has been particularly effective for elderly patients who were unable to bear the side effects of high-dose corticosteroids alone [52,53]. It is a folic acid analog that results in inhibition of dihydrofolate reductase enzyme. It also has anti-inflammatory properties and causes cytotoxicity [36,51].

The recommended dosage for methotrexate for BP treatment is 2.5–15 mg per week depending upon the requirements. A dose greater than 15 mg per week decreases its oral bioavailability [36]. It has, however, been stated that doses reaching 25 mg per week are also considered safe [51].

Contraindications for the use of methotrexate include a pregnant or breastfeeding state, severe hematologic derangements in cell counts (anemias, thrombocytopenia, and leukopenia), alcoholism, active infection, and severe liver or renal impairment [36]. The notable side effects include toxicities, especially those of GI, pulmonary, hematologic, and mucocutaneous [54].

Cyclophosphamide, an alkylating agent metabolized by CYP450, is an effective suppressor of both types of immunity – humoral and cell mediated. Its use has been documented by several case reports and small series for treatment of BP patients. Cyclophosphamide can be an excellent second-line option to be administered orally or intravenously when lower doses (50–100 mg/d) are used, especially in the older population [36]. Cyclophosphamide therapy is especially recommended in patients who develop serious side effects with corticosteroid therapy. The known side effects of cyclophosphamide include reversible alopecia and leukopenia [54]. Other adverse effects are potential subfertility due to azoospermia and/or ovarian insufficiency, SIADH, hemorrhagic cystitis (an indication for withdrawal), and cardiopulmonary toxicities [36].

Azathioprine and mycophenolate mofetil have also proved to be beneficial. Azathioprine (AZA) is derived from 6-mercaptopurine and has dual anti-inflammatory and immunosuppressive effects. It should be noted that there have been no significant variations in recorded response rates in patients who received AZA as an adjunct treatment in comparison to those treated with received systemic corticosteroids solely [55,56]. Adverse effects commonly seen in patients include gastrointestinal discomfort and oral ulcers. Rare side effects include macrocytosis, bone marrow supression and consequent cytopenias, deranged liver functions, idiosyncratic hypersensitivity, pancreatitis, reversible hair loss, as well as suggested oncogenic potential [36]. Furthermore, patients with reduced activity of thiopurine methyltransferase (TMTP) enzyme are at a higher risk of developing marrow suppression; therefore the assessment TMTP levels before prescription of AZA are crucial [36,51].

Mycophenolate inhibits the DNA synthesis of nucleotides and is used similarly to AZA for the treatment of BP. Mycophenolate is considered as effective as AZA but with lesser side effects [36,51,57]. Enteric-coated mycophenolate sodium is preferred as it reduces GI intolerance.

### 3.5 Intravenous immunoglobulins (IVIG)

The role of IVIG treatment for BP has been suggested for drug-resistant BP. It has a low adverse effect profile, but is an expensive treatment modality [51,58]. This therapy has been proven to significantly lower the levels of pathologic antibodies in animal models [59]. The optimal recommended dose is 2 mg/kg/cycle for IVIG therapy [58]. The average duration of treatment consisted of 24 months and an average of 15 cycles were administered for each patient [51].

In 2017 a large, multicenter, clinical trial was carried out to determine the effect of high-dose IVIG on BP patients. 400 mg/kg/day was administered for 5 days in patients who failed to improve with prednisolone (≥0.4 mg/kg/day). The results proved that IVIG was an effective treatment for steroid-resistant BP [60].

### 3.6 Therapeutic plasma exchange

Plasma exchange treatment for bullous pemphigoid is an exceptional adjunctive therapy, although its high cost limits its availability [36,61]. One of its primary benefits is that it allows for corticosteroid sparing in sick individuals [62]. As with any delicate procedure, plasmapheresis should be performed in specialized healthcare units to decrease the incidence of possible side effects including electrolyte derangements, infections and thromboembolic events [51]. It requires the formation of an arterio-venous fistula and can sometimes result in associated complications. Due to the rebound effect that is created by autoreactive lymphocyte activation following the exchange, the therapy is typically provided in combination with either systemic steroids or immunosuppressive agents, which allow for reduction of the effect [36].

Plasma exchange therapy is advantageous due to its ability to clear up existing auto-antibodies and cytokines from the circulatory system. As explained earlier, auto-antibodies are the chief inciting factor in the formation of skin lesions in BP. The rapid clinical response exhibited by patients is strongly suggestive of substantial amounts of auto-antibody clearance [63].

## 4 Emerging treatments

### 4.1 Avdoralimab

One of the new treatments for bullous pemphigoid is avdoralimab, a monoclonal antibody that specifically targets C5aR1. Previous studies have demonstrated the role of complement fragment C5a and C5a-C5aR interaction in mast cell degranulation, which causes formation of blisters in BP [64,65]. This has led to an interest in the use of C5 blocking agents for BP treatment. However, recent evidence suggests that inhibition of the C5a-C5aR2 axis, which although controversial, seems to serve a regulatory and protective role against the inflammatory actions of C5aR1 in BP patients [66,67]. Experiments by Karsten et al. showed that C5aR1 facilitated the pathological effects of C5a whereas C5aR2 exhibited a protective effect from blistering lesions. These experiments have also provided evidence that specific C5aR1 inhibition leads to reduced skin blistering [66,68].

With this rationale under consideration, a randomized, clinical trial in phase 2 is presently underway to examine the clinical effectiveness of avdoralimab for BP treatment in comparison with isolated topical steroid therapy. Patients will receive a regimen of 2 subcutaneous (SC) injections of avdoralimab every week for 12 weeks. The primary outcome is to evaluate the effectiveness through the proportion of patients who successfully achieve complete absence of bullous lesions in a period of 3 months without any relapse during the defined period.

### 4.2 Tildrakizumab

In BP patients, research has indicated the presence of interleukins 23 and 17 in blistering skin and elevated levels have helped identify patients who ultimately relapsed, hence hinting at potential targets for therapeutic agents [69,70]. Tildrakizumab, a humanized monoclonal antibody that targets p19 of IL-23 gained FDA approval in 2018 for use in plaque psoriasis after results from 3 randomized clinical trials demonstrated its effectiveness in improving skin lesions along with life quality for individuals with psoriasis [71,72]. The US FDA-approved dosing parameters are 100 mg SC injection at both week 0 and 4 after which a dose of 100 mg SC will be given every 12 weeks [73,74].

An open-label, clinical trial in early phase 1 (NCT04465292) is planned to test the efficacy of tildrakizumab in treating BP patients. Three doses of 100 mg will be dispensed at weeks 0, 4 and 16 with follow-up concluding at week 24. The primary outcome to be determined is a change in the severity of disease ranging from mild, moderate to severe based on number of lesions.

### 4.3 AKST4290

Prior studies have provided evidence of eotaxin and CCR3 involvement in the pathogenesis of BP [7,23,75]. AKST4290 is an oral drug that targets CCR3, which is a receptor for eotaxin. It has proven to be beneficial in trials for management of wet age-related macular degeneration without any significant side effects [76]. A similar drug, bertilimumab, an eotaxin-1 antagonist, has already proven to be efficacious for BP treatment (NCT02226146) and has gained FDA approval [6].

A phase 2, double-blind, clinical trial testing AKST4290's therapeutic effect in BP patients has been completed (NCT04499235). Thirty participants were enrolled to receive AKST4290 400 mg twice daily along with mometasone furoate until existing lesions healed and there were ≤3 new lesions/day. The primary outcome was an evaluation of the proportion of BP patients that successfully achieved control of disease without requiring rescue therapy. No results have been posted as of yet.

### 4.4 Benralizumab

IL-5 concentrations have been found in increasing quantities in blister fluid of active disease in BP patients and have been linked to the recruitment of eosinophils and their pathogenic role in BP [15,77,78]. Benralizumab is a humanized monoclonal antibody which targets IL-5Ra and has been involved in directly causing eosinophil apoptosis whereas other IL-5 agents have shown a reduction of eosinophil binding with IL-5 [79,80]. It gained FDA approval in 2017 for the management of severe eosinophilic asthma due to its documented ability to reduce rates of exacerbation in asthmatic patients [80]. The dosage regime used in trials is 30 mg every 4 weeks for 3 doses and then every 8 weeks [80,81]. It appeared to be well tolerated.

A randomized clinical trial in phase 3 is currently underway to assess the effectiveness of benralizumab therapy in management of BP patients (NCT04612790). Benralizumab will be administered subcutaneously as a loading dose followed by repeated dosing of benralizumab along with oral steroids that will gradually be tapered. The primary outcome is to evaluate the proportion of patients in complete remission who have discontinued oral steroid therapy for more than 2 months at week 36.

### 4.5 Dupilumab

Dupilumab targets IL-4Rα, which is a common subunit for both interleukins 4 and 13, hence inhibiting cytokine signaling [82,83]. It is a monoclonal antibody that is fully human. In 2017, it received approval for moderate and severe atopic dermatitis management but has since been shown to be efficacious against a variety of dermatological conditions [84].

The atopic dermatitis dosing regimen has been used most commonly: 600 mg SC loading dose and subsequent dosing of 300 mg SC every other week with an increase in the frequency of dosing as needed [[85], [86], [87]]. Results from the largest multicenter case series by Abdat et al. were encouraging, with 7 out of 13 patients achieving absolute resolution of itching and bullae formation and an additional 5 reporting clinical improvement with a willingness to continue the drug. The response time varied from 1 to 5 months with the median lying at 2 months. No adverse effects were noted.

There is also increasing evidence of dupilumab's ability to improve pruritis, which is a major concern in BP patients [86,88]. The effect seems to stem from its attenuation of sensory itch signals that are driven by interleukins 4, 13 and eosinophils [82,89]. This is a major benefit for patients suffering from intractable pruritus who are refractory to conventional therapeutic agents and omalizumab [88].

A phase 2/3 clinical trial (NCT04206553) is presently ongoing to establish the effectiveness of dupilumab in attaining continuous remission off oral steroids in BP patients. Patients will be administered a loading subcutaneous dose of dupilumab followed by one subcutaneous dose every 2 weeks. The chief endpoint is the proportion of BP patients who are successful in achieving sustained remission till week 36.

## 5 Conclusion

Research efforts to secure novel therapies for bullous pemphigoid are well underway due to the ample, unmet need in the field. While topical steroids have been described as first-line therapeutics, the safety profiles and potent effects of a multitude of potential agents currently being studied offer exciting alternatives for the treatment of elderly patients. Our knowledge regarding the pathogenesis of BP has increased greatly in the last few years, leading to the development of newer targets for therapy. In addition to the latest clinical trials discussed in this review and previous ones [6], future approaches are being considered towards the development of antigenic specific immunoadsorption for BP and the CAAR-T-cell approach, which could be customized according to the individual's antibodies to provide a personalized treatment regimen [90]. The extensive translational research being employed for BP treatment can potentially provide a new therapeutic horizon.

## 6 Limitations and challenges

The major limitation of this review concerns the promising new therapeutics. Many of these drugs, while currently in trial for the treatment of BP, have no prior literature regarding their efficacy in BP patients. The only exception to this was dupilumab, which has shown promise in BP in many studies [[85], [86], [87], [88], [89]]. Nonetheless, as some drugs have been used for other atopic conditions, the rationale for including them in our review was derived from recent advancements in our understanding of BP and its manifestations. Specifically, there have been numerous studies regarding the pathogenesis of BP, which have provided a wealth of experimental data for hypothesis-driven studies for new therapeutics. The most recent review of current drug development for pemphigoid diseases was published in 2020 [90]. In a similar fashion, we reviewed the latest discoveries and highlighted drugs that showed promise according to their targets or mechanisms of action. As time will tell, the results of these trials may very well expand our arsenal of therapeutics against BP.

## Provenance and peer-review

Not commissioned, externally peer-reviewed.

## Disclosures

NA.

## Funding

NA.

## Ethical approval

NA.

## Consent

NA.

## Author contribution

SNK, ZAK: conceived the idea, designed the study, and drafted the manuscript.

MHA, TA: conducted literature search and created the illustrations.

TA, TK: revised the manuscript critically and gave the final approval.

Revised the paper: TK, VRN.

## Registration of research studies

1. Name of the registry: NA.

2. Unique Identifying number or registration ID: NA.

3. Hyperlink to your specific registration (must be publicly accessible and will be checked): NA.

## Guarantor

Talal Almas.

## Declaration of competing interest

NA.
